# 2-Methyl­propan-2-aminium 4-hy­droxy­benzoate

**DOI:** 10.1107/S1600536810022592

**Published:** 2010-06-18

**Authors:** Shu-Lan Yu

**Affiliations:** a Chemistry Engineering Department, Weifang Vocational College, Weifang 261000, People’s Republic of China

## Abstract

In the crystal of the title mol­ecular salt, C_4_H_12_N^+^·C_7_H_5_O_3_^−^, the cation is linked to three nearby anions by N—H⋯O hydrogen bonds. An O—H⋯O hydrogen bond between anions further consolidates the packing.

## Related literature

For a related structure, see: Scholz & Gorls (2002 ▶).
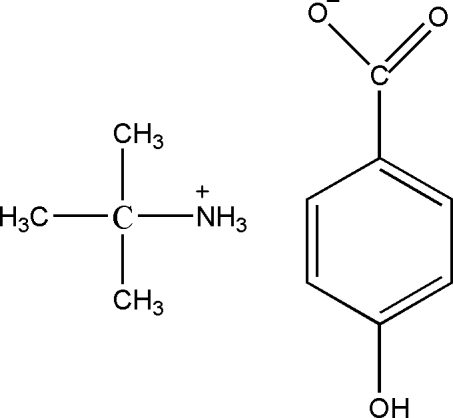


## Experimental

### 

#### Crystal data

C_4_H_12_N^+^·C_7_H_5_O_3_^−^
*M* *_r_* = 211.26
Monoclinic, 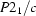

*a* = 6.8300 (14) Å
*b* = 9.2790 (19) Å
*c* = 19.831 (4) Å
β = 99.58 (3)°
*V* = 1239.3 (4) Å^3^
*Z* = 4
Mo *K*α radiation
μ = 0.08 mm^−1^
*T* = 293 K
0.10 × 0.09 × 0.08 mm

#### Data collection

Bruker SMART CCD diffractometer
2899 measured reflections
2677 independent reflections
1804 reflections with *I* > 2σ(*I*)
*R*_int_ = 0.039

#### Refinement

*R*[*F* ^2^ > 2σ(*F* ^2^)] = 0.057
*wR*(*F* ^2^) = 0.168
*S* = 1.04
2677 reflections
149 parameters
H atoms treated by a mixture of independent and constrained refinement
Δρ_max_ = 0.22 e Å^−3^
Δρ_min_ = −0.29 e Å^−3^

### 

Data collection: *SMART* (Bruker, 2003 ▶); cell refinement: *SAINT* (Bruker, 2003 ▶); data reduction: *SAINT*; program(s) used to solve structure: *SHELXS97* (Sheldrick, 2008 ▶); program(s) used to refine structure: *SHELXL97* (Sheldrick, 2008 ▶); molecular graphics: *SHELXTL* (Sheldrick, 2008 ▶); software used to prepare material for publication: *SHELXTL*.

## Supplementary Material

Crystal structure: contains datablocks global, I. DOI: 10.1107/S1600536810022592/hb5493sup1.cif

Structure factors: contains datablocks I. DOI: 10.1107/S1600536810022592/hb5493Isup2.hkl

Additional supplementary materials:  crystallographic information; 3D view; checkCIF report

## Figures and Tables

**Table 1:** Hydrogen-bond geometry (Å, °)

*D*—H⋯*A*	*D*—H	H⋯*A*	*D*⋯*A*	*D*—H⋯*A*
O3—H3*B*⋯O1^i^	0.82	1.83	2.621 (2)	163
N1—H1⋯O2^ii^	0.92 (2)	1.93 (2)	2.835 (2)	168.2 (18)
N1—H3⋯O2	0.94 (2)	1.93 (2)	2.842 (2)	162.2 (18)
N1—H2⋯O1^iii^	0.87 (2)	1.92 (3)	2.796 (2)	174.7 (19)

Symmetry codes: (i) 
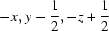
; (ii) 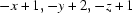; (iii) 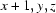.

**Fig. 1.**
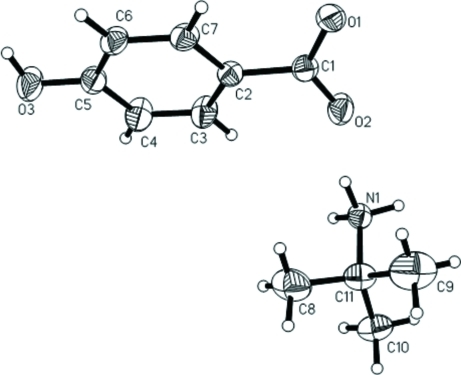
The molecular structure of (I) with displacement ellipsoids drawn at the 30% probability level.

## supplementary crystallographic information

### Experimental

A mixture of 2-methylpropan-2-amine(0.02 mol) and 4-hydroxybenzoic acid (0.02 mol) was stirred in ethanol (30 ml) at 353 K for 3 h to afford the title compound (yield 50%).  Colourless bars of (I) were obtained by recrystallization from acetone at room temperature.

### Refinement

H atoms were positioned geometrically and allowed to ride on their parent atoms, with N—H and C—H distances of 0.86 and 0.93–0.96 Å, respectively, and with *U*_iso_(H) = 1.2*U*_eq_ of the parent atoms.

### Crystal data

**Table:**

C_4_H_12_N^+^·C_7_H_5_O_3_^−^	*F*(000) = 456
*M**_r_* = 211.26	*D*_x_ = 1.132 Mg m^−^^3^
Monoclinic, *P*2_1_/*c*	Mo *K*α radiation, λ = 0.71073 Å
Hall symbol: -P 2ybc	Cell parameters from 1804 reflections
*a* = 6.8300 (14) Å	θ = 2.1–27.0°
*b* = 9.2790 (19) Å	µ = 0.08 mm^−^^1^
*c* = 19.831 (4) Å	*T* = 293 K
β = 99.58 (3)°	Bar, colorless
*V* = 1239.3 (4) Å^3^	0.10 × 0.09 × 0.08 mm
*Z* = 4	

### Data collection

**Table:**

Bruker SMART CCD diffractometer	1804 reflections with *I* > 2σ(*I*)
Radiation source: fine-focus sealed tube	*R*_int_ = 0.039
graphite	θ_max_ = 27.0°, θ_min_ = 2.1°
phi and ω scans	*h* = 0→8
2899 measured reflections	*k* = 0→11
2677 independent reflections	*l* = −23→23

### Refinement

**Table:**

Refinement on *F*^2^	Secondary atom site location: difference Fourier map
Least-squares matrix: full	Hydrogen site location: inferred from neighbouring sites
*R*[*F*^2^ > 2σ(*F*^2^)] = 0.057	H atoms treated by a mixture of independent and constrained refinement
*wR*(*F*^2^) = 0.168	*w* = 1/[σ^2^(*F*_o_^2^) + (0.0928*P*)^2^ + 0.1735*P*] where *P* = (*F*_o_^2^ + 2*F*_c_^2^)/3
*S* = 1.04	(Δ/σ)_max_ < 0.001
2677 reflections	Δρ_max_ = 0.22 e Å^−^^3^
149 parameters	Δρ_min_ = −0.29 e Å^−^^3^
0 restraints	Extinction correction: *SHELXL97* (Sheldrick, 2008), Fc^*^=kFc[1+0.001xFc^2^λ^3^/sin(2θ)]^-1/4^
Primary atom site location: structure-invariant direct methods	Extinction coefficient: 0.59 (3)

### Special details

**Table:**

Geometry. All e.s.d.'s (except the e.s.d. in the dihedral angle between two l.s. planes) are estimated using the full covariance matrix. The cell e.s.d.'s are taken into account individually in the estimation of e.s.d.'s in distances, angles and torsion angles; correlations between e.s.d.'s in cell parameters are only used when they are defined by crystal symmetry. An approximate (isotropic) treatment of cell e.s.d.'s is used for estimating e.s.d.'s involving l.s. planes.
Refinement. Refinement of *F*^2^ against ALL reflections. The weighted *R*-factor *wR* and goodness of fit *S* are based on *F*^2^, conventional *R*-factors *R* are based on *F*, with *F* set to zero for negative *F*^2^. The threshold expression of *F*^2^ > σ(*F*^2^) is used only for calculating *R*-factors(gt) *etc*. and is not relevant to the choice of reflections for refinement. *R*-factors based on *F*^2^ are statistically about twice as large as those based on *F*, and *R*- factors based on ALL data will be even larger.

### Fractional atomic coordinates and isotropic or equivalent isotropic displacement parameters (Å^2^)

**Table:**

	*x*	*y*	*z*	*U*_iso_*/*U*_eq_	
C1	0.1770 (3)	0.81042 (19)	0.40216 (9)	0.0475 (4)	
C2	0.2054 (2)	0.70697 (18)	0.34696 (8)	0.0435 (4)	
C3	0.3911 (3)	0.6882 (2)	0.32850 (10)	0.0618 (6)	
H3A	0.4999	0.7372	0.3523	0.074*	
C4	0.4170 (3)	0.5976 (3)	0.27512 (12)	0.0728 (7)	
H4A	0.5423	0.5872	0.2632	0.087*	
C5	0.2577 (3)	0.5225 (2)	0.23940 (10)	0.0571 (5)	
C6	0.0722 (3)	0.5367 (2)	0.25849 (10)	0.0558 (5)	
H6A	−0.0351	0.4843	0.2359	0.067*	
C7	0.0470 (2)	0.6290 (2)	0.31113 (9)	0.0513 (5)	
H7A	−0.0785	0.6393	0.3229	0.062*	
O1	0.00062 (19)	0.84869 (15)	0.40644 (6)	0.0606 (4)	
O2	0.3246 (2)	0.85859 (16)	0.44198 (7)	0.0667 (5)	
O3	0.2922 (2)	0.4377 (2)	0.18682 (9)	0.0863 (6)	
H3B	0.1865	0.4187	0.1622	0.129*	
C8	0.7450 (4)	0.6075 (3)	0.50722 (16)	0.0949 (9)	
H8A	0.6109	0.6040	0.4832	0.142*	
H8B	0.7693	0.5259	0.5372	0.142*	
H8C	0.8352	0.6053	0.4749	0.142*	
C9	0.6313 (5)	0.7601 (4)	0.59955 (17)	0.1178 (12)	
H9A	0.6486	0.8522	0.6218	0.177*	
H9B	0.6563	0.6850	0.6331	0.177*	
H9C	0.4977	0.7520	0.5754	0.177*	
C10	0.9911 (4)	0.7600 (3)	0.58411 (13)	0.0806 (7)	
H10A	1.0103	0.8519	0.6065	0.121*	
H10B	1.0772	0.7522	0.5507	0.121*	
H10C	1.0213	0.6845	0.6173	0.121*	
C11	0.7764 (3)	0.7461 (2)	0.54907 (11)	0.0632 (6)	
N1	0.7346 (2)	0.86993 (18)	0.49897 (8)	0.0483 (4)	
H1	0.734 (3)	0.958 (2)	0.5202 (10)	0.058*	
H2	0.815 (3)	0.869 (2)	0.4690 (12)	0.058*	
H3	0.608 (3)	0.859 (2)	0.4718 (10)	0.058*	

### Atomic displacement parameters (Å^2^)

**Table:**

	*U*^11^	*U*^22^	*U*^33^	*U*^12^	*U*^13^	*U*^23^
C1	0.0461 (10)	0.0530 (10)	0.0442 (8)	0.0012 (8)	0.0095 (7)	0.0012 (7)
C2	0.0394 (9)	0.0475 (9)	0.0439 (8)	−0.0014 (7)	0.0077 (7)	0.0004 (7)
C3	0.0401 (10)	0.0804 (13)	0.0662 (11)	−0.0155 (9)	0.0120 (8)	−0.0224 (10)
C4	0.0406 (10)	0.1022 (17)	0.0781 (14)	−0.0061 (10)	0.0176 (9)	−0.0322 (12)
C5	0.0449 (10)	0.0678 (12)	0.0576 (10)	0.0055 (8)	0.0055 (8)	−0.0158 (9)
C6	0.0394 (9)	0.0673 (12)	0.0583 (10)	−0.0050 (8)	0.0008 (7)	−0.0132 (9)
C7	0.0356 (8)	0.0661 (11)	0.0523 (9)	−0.0023 (8)	0.0070 (7)	−0.0036 (8)
O1	0.0513 (8)	0.0813 (10)	0.0505 (7)	0.0161 (7)	0.0122 (6)	−0.0023 (6)
O2	0.0521 (8)	0.0788 (10)	0.0674 (9)	−0.0015 (7)	0.0051 (6)	−0.0267 (7)
O3	0.0537 (8)	0.1141 (13)	0.0890 (11)	0.0104 (8)	0.0058 (7)	−0.0525 (10)
C8	0.0918 (18)	0.0576 (14)	0.127 (2)	−0.0112 (12)	−0.0065 (16)	0.0126 (14)
C9	0.098 (2)	0.161 (3)	0.107 (2)	0.013 (2)	0.0542 (18)	0.056 (2)
C10	0.0671 (14)	0.0876 (16)	0.0802 (15)	0.0001 (12)	−0.0078 (12)	0.0154 (13)
C11	0.0545 (11)	0.0673 (12)	0.0678 (12)	−0.0024 (10)	0.0105 (9)	0.0151 (10)
N1	0.0421 (8)	0.0528 (9)	0.0510 (8)	−0.0009 (7)	0.0108 (7)	−0.0036 (7)

### Geometric parameters (Å, °)

**Table:**

C1—O2	1.255 (2)	C8—H8A	0.9600
C1—O1	1.272 (2)	C8—H8B	0.9600
C1—C2	1.493 (2)	C8—H8C	0.9600
C2—C3	1.388 (2)	C9—C11	1.527 (3)
C2—C7	1.394 (2)	C9—H9A	0.9600
C3—C4	1.386 (3)	C9—H9B	0.9600
C3—H3A	0.9300	C9—H9C	0.9600
C4—C5	1.384 (3)	C10—C11	1.520 (3)
C4—H4A	0.9300	C10—H10A	0.9600
C5—O3	1.358 (2)	C10—H10B	0.9600
C5—C6	1.388 (3)	C10—H10C	0.9600
C6—C7	1.383 (3)	C11—N1	1.515 (2)
C6—H6A	0.9300	N1—H1	0.92 (2)
C7—H7A	0.9300	N1—H2	0.87 (2)
O3—H3B	0.8200	N1—H3	0.94 (2)
C8—C11	1.527 (3)		
			
O2—C1—O1	121.95 (16)	H8A—C8—H8C	109.5
O2—C1—C2	120.11 (16)	H8B—C8—H8C	109.5
O1—C1—C2	117.93 (15)	C11—C9—H9A	109.5
C3—C2—C7	117.80 (15)	C11—C9—H9B	109.5
C3—C2—C1	120.66 (15)	H9A—C9—H9B	109.5
C7—C2—C1	121.53 (15)	C11—C9—H9C	109.5
C4—C3—C2	121.05 (17)	H9A—C9—H9C	109.5
C4—C3—H3A	119.5	H9B—C9—H9C	109.5
C2—C3—H3A	119.5	C11—C10—H10A	109.5
C5—C4—C3	120.47 (18)	C11—C10—H10B	109.5
C5—C4—H4A	119.8	H10A—C10—H10B	109.5
C3—C4—H4A	119.8	C11—C10—H10C	109.5
O3—C5—C4	117.57 (17)	H10A—C10—H10C	109.5
O3—C5—C6	123.22 (17)	H10B—C10—H10C	109.5
C4—C5—C6	119.21 (17)	N1—C11—C10	107.33 (16)
C7—C6—C5	119.93 (16)	N1—C11—C8	106.78 (18)
C7—C6—H6A	120.0	C10—C11—C8	110.92 (19)
C5—C6—H6A	120.0	N1—C11—C9	107.08 (19)
C6—C7—C2	121.50 (16)	C10—C11—C9	112.0 (2)
C6—C7—H7A	119.3	C8—C11—C9	112.4 (2)
C2—C7—H7A	119.3	C11—N1—H1	113.0 (13)
C5—O3—H3B	109.5	C11—N1—H2	111.4 (13)
C11—C8—H8A	109.5	H1—N1—H2	111.9 (19)
C11—C8—H8B	109.5	C11—N1—H3	110.4 (12)
H8A—C8—H8B	109.5	H1—N1—H3	106.4 (18)
C11—C8—H8C	109.5	H2—N1—H3	103.3 (18)

### Hydrogen-bond geometry (Å, °)

**Table:**

*D*—H···*A*	*D*—H	H···*A*	*D*···*A*	*D*—H···*A*
O3—H3B···O1^i^	0.82	1.83	2.621 (2)	163
N1—H1···O2^ii^	0.92 (2)	1.93 (2)	2.835 (2)	168.2 (18)
N1—H3···O2	0.94 (2)	1.93 (2)	2.842 (2)	162.2 (18)
N1—H2···O1^iii^	0.87 (2)	1.92 (3)	2.796 (2)	174.7 (19)

Symmetry codes: (i) −*x*, *y*−1/2, −*z*+1/2; (ii) −*x*+1, −*y*+2, −*z*+1; (iii) *x*+1, *y*, *z*.
